# P02.95. Treating type 2 diabetes: a cross-sectional audit of naturopathic standards of care using the Naturopathic Patient Database

**DOI:** 10.1186/1472-6882-12-S1-P151

**Published:** 2012-06-12

**Authors:** C Habib, S Podgrabinski, M Gowan, K Cooley

**Affiliations:** 1 Canadian College of Naturopathic Medicine, Toronto, Canada

## Purpose

The Naturopathic Patient Database (NPD) is a data management tool developed by the Canadian College of Naturopathic Medicine (CCNM) to collect patient data from its teaching clinic, the Robert Schad Naturopathic Clinic (RSNC). This database was created in 2006 and has evolved to meet the various academic and research needs of CCNM. This study investigates how type 2 diabetes mellitus (T2DM) is managed at the RSNC from May 2009 to present.

## Methods

Cases of T2DM from the RSNC reported in the NPD were extracted based on an ICD-10 code assessment of E11 (non-insulin-dependant diabetes mellitus). One auditor reviewed 30 files and tabulated audit scores. The Research Ethics Board of CCNM provided ethical oversight of this project. The American Diabetes Association 2010 standards of medical care in diabetes were used as guidelines for the audit. Multiple categories in diagnosis, physical exam, labs, and management were graded on a 0-2 scale. The Measure Yourself Medical Outcome Profile (MYMOP) is used by the RSNC as a universal outcome measure of effectiveness of individualized patient-defined symptoms and was incorporated into the audit and reporting of results.

## Results

The average audit score is 55.5/90. The most common interventions being used are diet and aerobic exercise, followed by supplements (omega-3 fatty acids) and botanicals.

## Conclusion

Preliminary data suggests that the standards of care for T2DM are not followed stringently, particularly with regards to complete physical exams, appropriate referrals, and goal-setting. Education and creation of a naturopathic standard of care may improve audit performance and patient outcomes.

